# Effect of Omega-3 Fatty Acid Supplementation on Oxylipins in a Routine Clinical Setting

**DOI:** 10.3390/ijms19010180

**Published:** 2018-01-08

**Authors:** Christoph Schmöcker, Ingrid W. Zhang, Stefanie Kiesler, Ursula Kassner, Annika I. Ostermann, Elisabeth Steinhagen-Thiessen, Nils H. Schebb, Karsten-H. Weylandt

**Affiliations:** 1 Medical Department, Division of Gastroenterology, Oncology, Hematology, Rheumatology and Diabetes, Ruppiner Kliniken, Brandenburg Medical School, 16816 Neuruppin, Germany; ChrisSchmoecker@web.de (C.S.); S.Kiesler@ruppiner-kliniken.de (S.K.); 2 Department of Gastroenterology, Sana Klinikum Lichtenberg, 10365 Berlin, Germany; 3 Medical Department, Division of Gastroenterology and Nephrology, Campus Virchow-Klinikum, Charité University Medicine, 13353 Berlin, Germany; ingrid-wei.zhang@charite.de (I.W.Z.); Ursula.Kassner@charite.de (U.K.); Elisabeth.Steinhagen-Thiessen@charite.de (E.S.-T.); 4 Faculty of Mathematics and Natural Sciences, University of Wuppertal, 42119 Wuppertal, Germany; annika.ostermann@schebb-web.de (A.I.O.); nils@schebb-web.de (N.H.S.); 5 Institute for Food Toxicology, University for Veterinary Medicine Hannover, 30559 Hannover, Germany

**Keywords:** omega-3 fatty acids, oxylipins, lipidomics, lipid clinic, hyperlipidemia

## Abstract

Omega-6 polyunsaturated fatty acid (*n*-6 PUFA) is the predominant polyunsaturated fatty acid (PUFA), especially in Western diet. A high omega-6/omega-3 ratio in Western diets is implicated in the development of cardiovascular diseases and inflammatory processes. Studies in animal models and in humans have demonstrated beneficial effects of omega-3 PUFA (*n*-3 PUFA) in a variety of diseases, including cardiac arrhythmias and inflammatory diseases, as well as breast and colon cancer. The molecular mechanisms underlying the effects of *n*-3 PUFA are still not well understood. Possible mechanisms include competition between *n*-3 and *n*-6 PUFAs at the cyclooxygenase (COX) and lipoxygenase (LOX) and cytochrome P450 levels, and subsequent formation of oxylipins with specific anti-inflammatory or anti-arrhythmic effects. In this study, we report the impact of routine long-term treatment with prescription-grade *n*-3 PUFA (either 840 mg or 1680 mg per day) on blood cell membrane fatty acid composition, as well as plasma oxylipin patterns, in a patient population with severe hyperlipidemia and cardiovascular disease who are on standard lipid-lowering and cardioprotective medications. Lipidomics analyses were performed by LC/ESI-MS/MS. Supplementation led to a dose-dependent increase in *n*-3 PUFA eicosapentaenoic acid (EPA) and docosahexaenoic acid (DHA) in the blood cell fraction. We also observed a dose-dependent increase in EPA- and DHA-derived epoxy metabolites, whereas the effect of *n*-3 PUFA supplementation on LOX-dependent EPA- and DHA-derived hydroxy metabolites was less pronounced, with a tendency towards lower metabolites in subjects with higher *n*-3 PUFA levels. These data thus generally confirm effects of *n*-3 PUFA supplementation observed previously in healthy individuals. Additionally, they indicate a suppressive effect of high *n*-3 PUFA supplementation on the formation of LOX metabolites in the context of concomitant aspirin medication.

## 1. Introduction

Omega-6 polyunsaturated fatty acids (*n*-6 PUFAs) such as arachidonic acid (AA, 20:4 *n*-6) have been shown to enhance predominantly negative effects: pro-inflammatory [1], pro-thrombotic and pro-arrhythmogenic effects [2]. In contrast, epidemiologic as well as clinical studies support the hypothesis that omega-3 polyunsaturated fatty acids (*n*-3 PUFAs) from fish fat exert beneficial effects regarding cardiovascular disease and inflammatory processes such as rheumatoid arthritis and bronchial asthma. An elevated intake of *n*-3 PUFAs such as eicosapentaenoic acid (EPA, 20:5 *n*-3) and docosahexaenoic acid (DHA, 22:6 *n*-3) reduces the risk of sudden death from coronary artery disease, which is attributed to their antiarrhythmic effects [3]. Furthermore, *n*-3 PUFAs increase systemic arterial compliance in hyperlipidemic patients [4], lower blood pressure in a dose-dependent manner [5], and decrease triglyceride levels [6].

PUFAs are oxygenated by different enzymes such as cyclooxygenase (COX), lipoxygenase (LOX), and cytochrome P450 enzymes. Taken together with non-enzymatic pathways, a broad variety of bioactive lipid mediators can be generated, which are important for understanding the molecular mechanisms underlying the effects of *n*-3 PUFAs. Many of the EPA- and DHA-derived metabolites have been characterized and have been shown to have potent anti-inflammatory effects in a variety of inflammation models [7]. These include reduced platelet adherence and vasodilation by modulating transcription factors such as sterol regulatory-element binding protein (SREBP) [8] and the peroxisome proliferator-activated receptor (PPAR) [9]. In addition, an established view is that *n*-3 PUFA and its derivatives compete with AA at the enzymatic level, resulting in the generation of less inflammatory metabolites. Epoxydocosapentaenoic acids (EDPs) for instance, cytochrome P450 epoxygenase metabolites of DHA, have been reported to be more potent vasodilatory and anti-inflammatory compounds than AA-derived epoxyeicosatrienoic acids (EETs) [10].

Supplementing healthy subjects with EPA and DHA resulted in a marked increase of EPA- and DHA-derived lipid metabolites, emphasizing the notion that the production of oxylipins and eicosanoids can be influenced by the administration of *n*-3 PUFAs in healthy volunteers [11,12,13,14,15,16]. However, since the pharmaceutical intake of *n*-3 PUFA is an established treatment for patients with hypertriglyceridemia [17] and is also approved as a secondary prevention after myocardial infarction [18,19], it is of particular interest to assess the effect of *n*-3 PUFA supplementation on the plasma lipidome in the context of co-treatment with other cardiovascular drugs; in particular, aspirin and statins have been shown to modify lipid mediator formation from long-chain PUFAs [7,20,21,22].

In this study, we report the impact of long-term treatment with prescription-grade *n*-3 PUFAs (either 840 mg/day containing 460 mg EPA and 380 mg DHA as ethyl esters or twice the amount) on blood cell fatty acid composition, as well as plasma oxylipin patterns in a patient population with severe atherosclerosis and hyperlipidemia who are on long-term treatment with aspirin and lipid-lowering drugs.

## 2. Results

### 2.1. Characteristics of the Participants

All patients examined in this study were undergoing regular lipoprotein apheresis for severe dyslipidemia such as hypercholesterolemia, hyperlipoproteinemia (a), and combined hyperlipidemia. Samples were taken from individual subjects directly before the weekly apheresis session in order to reduce any effects on lipid metabolite formation due to apheresis. Sixteen patients, of which one was female, were included in the control group without *n*-3 PUFA supplementation. Mean BMI (body mass index) of this group was 28.9 (24.1–35.5) kg/m^2^. The low *n*-3 PUFA treatment group, which received 840 mg *n*-3 PUFA per day, included 11 patients with two of them being female. The mean BMI of this group was 30.4 (25.4–39.2) kg/m^2^. The third group was treated with 1680 mg *n*-3 PUFA per day and included eight patients (one of them female) with a mean BMI of 27.8 (22.3–30.3) kg/m^2^. The duration of *n*-3 PUFA supplementation was at least three months in all patients; thus, data represents steady-state values. Every patient in this study was on long-term medication with aspirin 100 mg per day. Most patients received statins; for further details see Table 1. Patients treated with *n*-3 PUFA had higher levels of triglycerides at the beginning, possibly reflecting the reason to initiate *n*-3 PUFA therapy in the first place. No statistically significant differences in the levels of high-density lipoprotein (HDL), low-density lipoprotein (LDL), and total cholesterol were observed among the groups.

### 2.2. Effect of n-3 PUFA Intake on Fatty Acid Composition in Blood Cells

**Figure 1:**
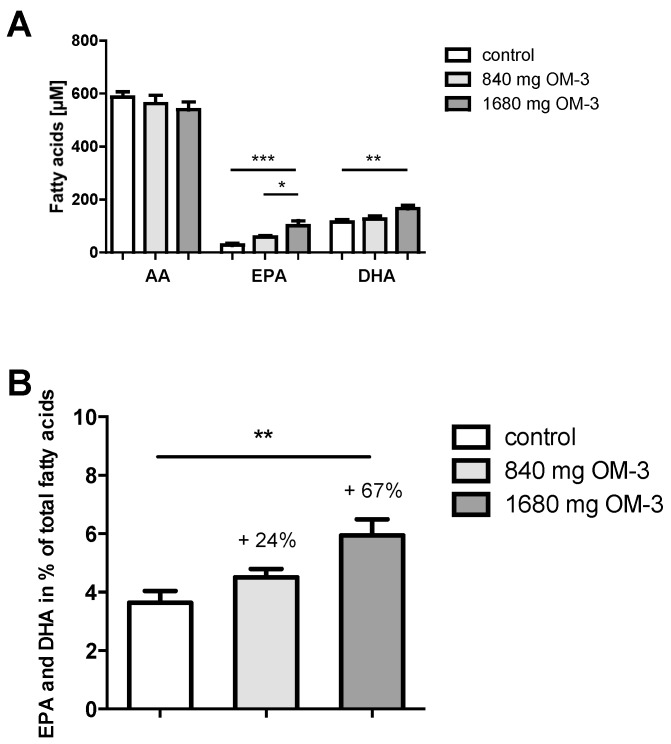
Omega-3 polyunsaturated fatty acids (*n*-3 PUFAs, shown as OM-3 in all figures) change the fatty acid composition in the blood cell fraction and affect the percentage of docosahexaenoic acid (DHA) and eicosapentaenoic acid (EPA) of total fatty acids in hyperlipidemic subjects. (**A**) Arachidonic acid (AA), EPA, and DHA levels in the control group and patients with 840 and 1680 mg *n*-3 PUFA supplementation; (**B**) Effect of *n*-3 PUFA supplementation on the percentage of DHA and EPA as a percentage of the total fatty acids in the blood cell fraction. The change compared to the group not receiving *n*-3 PUFA is also indicated as percentage change. All results are shown as the mean ± SEM. Differences between the groups are significant as indicated (* *p* < 0.05; ** *p* < 0.01; *** *p* < 0.001).

The fatty acid composition was measured in the blood cell fraction (predominantly erythrocyte membranes) as this reflects long-term intake of various fatty acids in the most appropriate manner [23]. Throughout the three different groups, palmitic acid (C16:0) was the most abundant fatty acid. The intake of 1680 mg *n*-3 PUFA led to a significant increase in EPA and DHA, while the intake of 840 mg *n*-3 PUFA did not increase levels significantly (Figure 1A). The level of AA measured was slightly reduced in both *n*-3 PUFA treatment groups. Supplementation with EPA/DHA led to an increase in the percentage of EPA+DHA of total fatty acids in the blood cell fraction (Figure 1B) in a dose-dependent manner (3.6% in the control group, 4.5% with 840 mg, and 6.0% with 1680 mg *n*-3 PUFA). A comprehensive list of the quantified fatty acids is shown in Supplementary Table S1.

### 2.3. Effect of n-3 PUFA Treatment on Plasma Levels of 13-HDHA and 18-HEPE

**Figure 2:**
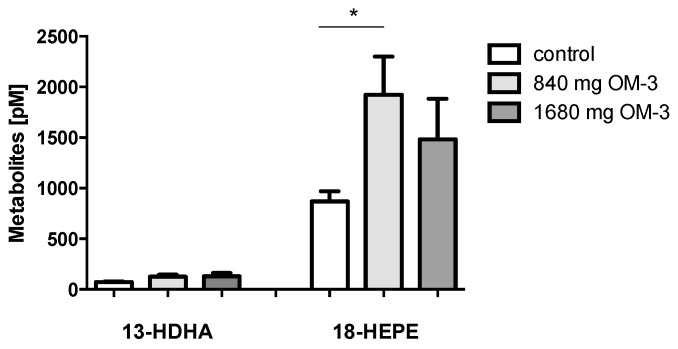
Effect of EPA/DHA supplementation on 18-hydroxyeicosapentaenoic acid (18-HEPE) and 13-hydroxydocohexaenoic acid (13-HDHA). 18-HEPE and 13-HDHA were measured in plasma of hyperlipidemic patients with different doses of *n*-3 PUFA as indicated. All results are shown as the mean ± SEM. Differences between the groups are significant as indicated (* *p* < 0.05).

18-Hydroxyeicosapentaenoic acid (18-HEPE) is an EPA-derived metabolite and the precursor of the anti-inflammatory E-resolvin family. It is generated through several mechanisms including conversion by autoxidation, by COX-2 [24], and by cytochrome P450 monooxygenases. The level of 18-HEPE in plasma of hyperlipidemic patients in the control group was 869.33 ± 101.5 pmol/L. Treatment with 840 mg *n*-3 PUFA resulted in a 2.2-fold elevation of 18-HEPE. This effect could not be enhanced by doubling the daily dose of *n*-3 PUFA (Figure 2). 13-Hydroxydocohexaenoic acid (13-HDHA) might be another biologically important compound. Recently, 13-hydroxy compounds derived from *n*-3 docosapentaenoic acid (DPA) were described as potent anti-inflammatory compounds triggered by atorvastatin treatment. 13-HDHA is generated by autoxidation of DHA and by the activity of COX-2 in the absence of aspirin [22]. In analogy, the formation of 13-HDHA might be increased by atorvastatin treatment [20]. However, in the patient samples analyzed here, 13-HDHA was not significantly altered by supplementation with EPA and DHA (Figure 2), and there was also no effect in the subgroup of atorvastatin-treated patients.

### 2.4. Changes in the Profile of LOX-Dependent AA-, EPA-, and DHA-Derived Metabolites

Hydroxy and hydroperoxy compounds are formed from PUFAs by the action of lipoxygenases. These are designated according to their ability to insert molecular oxygen at different carbon positions (5, 8, 12, or 15) of the polyunsaturated fatty acids.

**Figure 3:**
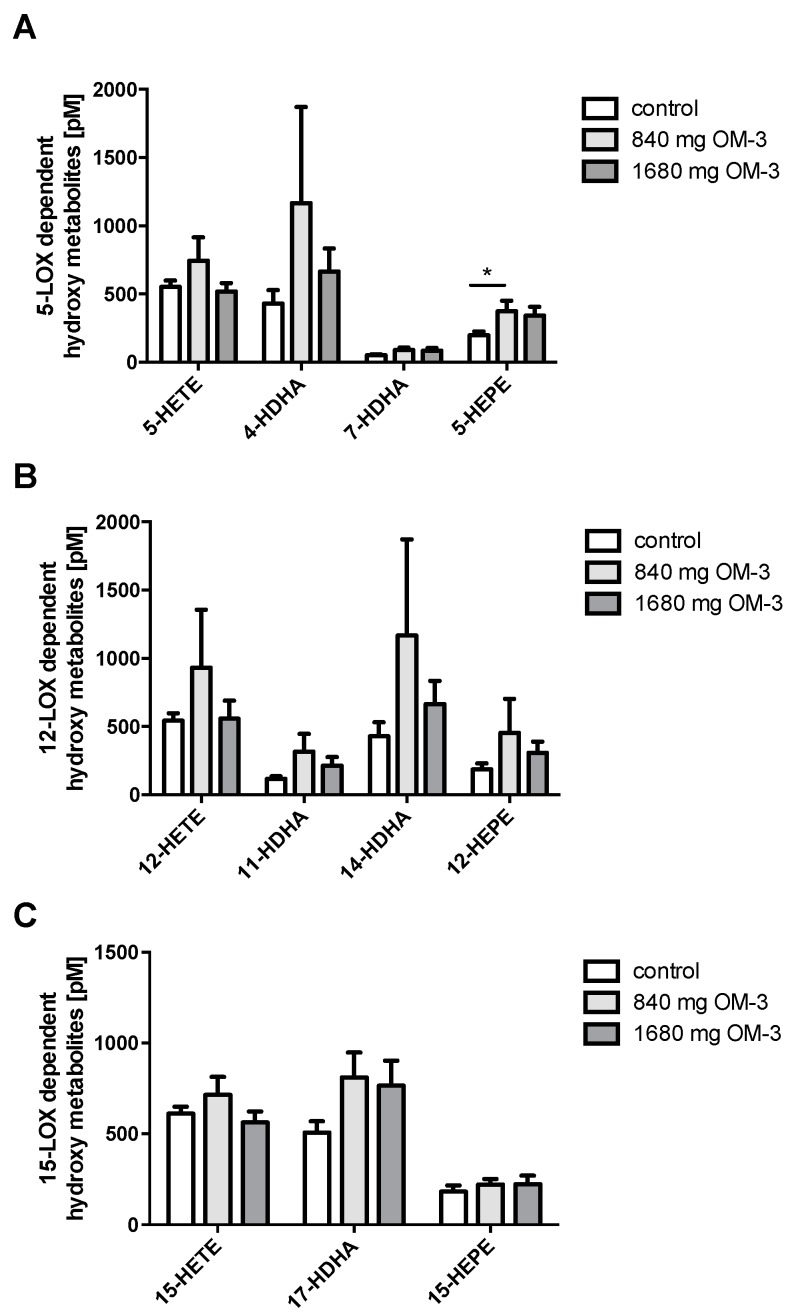
Effect of *n*-3 PUFA supplementation on 5-, 12-, and 15- lipoxygenase (LOX)-dependent AA-, DHA-, and EPA-derived hydroxy metabolites measured in plasma. (**A**) Changes in 5-LOX-dependent hydroxy metabolites; (**B**) Changes in 12-LOX-dependent hydroxy metabolites; (**C**) Changes in 15-LOX-dependent hydroxy metabolites. All results are shown as the mean ± SEM. Differences between the groups are significant as indicated (* *p* < 0.05).

AA-derived 5-LOX-dependent 5-HETE did not change in either of the *n*-3 PUFA treatment groups. In contrast, a 1.9-fold increase in 5-HEPE, a 5-LOX-dependent hydroxy metabolite of EPA, could be observed in the group taking 840 mg/day *n*-3 PUFA. This increase was not observed with the higher dose of *n*-3 PUFA, though. The plasma concentration of 4-HDHA, which exerts anti-inflammatory and anti-angiogenic effects [25], was 5.1 to 5.7 times higher than the concentration of 7-HDHA throughout the groups. Both 4-HDHA and 7-HDHA are 5-LOX-dependent hydroxy metabolites of DHA. However, in contrast to 5-HEPE, the levels of 4-HDHA and 7-HDHA did not change significantly by *n*-3 PUFA supplementation at either dose (Figure 3A).

12-LOX metabolizes AA to 12-HETE and converts EPA to 12-HEPE, and DHA to 11-HDHA and 14-HDHA. In this study, supplementation with *n*-3 PUFA did not change levels of 12-LOX-dependent AA-derived metabolites or levels of EPA- and DHA-derived metabolites (Figure 3B).

*n*-3 PUFA treatment also did not have any significant impact on 15-LOX-dependent AA-, EPA- and DHA-derived hydroxy metabolites such as 15-HETE, 15-HEPE, and 17-HDHA (Figure 3C). 17-HDHA, the precursor of the anti-inflammatory D-resolvin family, is formed by hydroxylation of DHA at C17 via 15-lipoxygenase (15-LOX) or via autoxidation. The mean amount of 17-HDHA in the control group was 507.29 ± 61.5 pmol/L and increased with the supplementation of *n*-3 PUFA (810.03 ± 138.2 and 764.86 ± 138.5 pmol/L with supplementation of 840 and 1680 mg EPA/DHA, respectively). In contrast to 18-HEPE, the other resolvin precursor, the plasma levels of 17-HDHA did not increase to a significant degree on *n*-3 PUFA treatment.

### 2.5. Effect of EPA and DHA Supplementation on AA-, DHA-, and EPA-Derived Epoxy Metabolites

**Figure 4:**
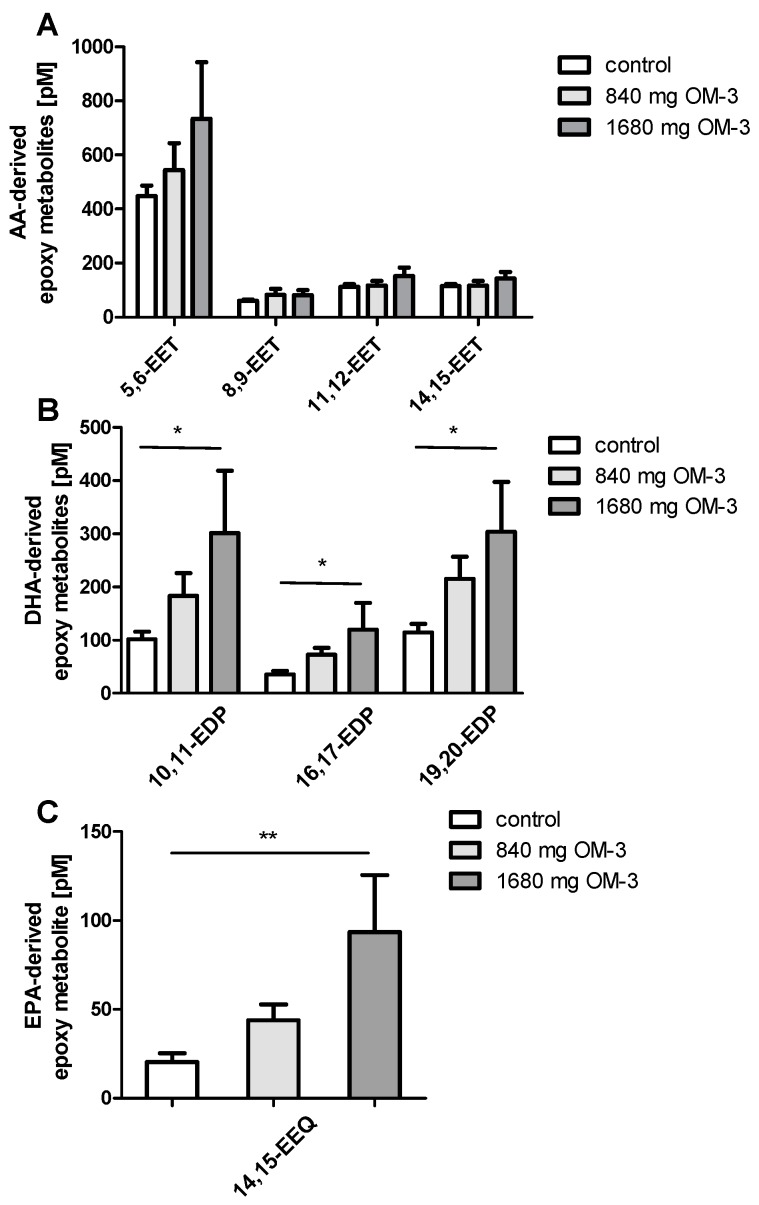
Comparison of AA-, DHA-, and EPA-derived epoxy metabolites. (**A**) Epoxyeicosatrienoic acids (EETs), AA-derived epoxy metabolites, were measured in plasma of hyperlipidemic patients receiving different doses of dietary *n*-3 PUFAs; (**B**,**C**) Accordingly, epoxydocosapentaenoic acids (EDPs) and 14,15-epoxyeicosatetraenoic acid (14,15-EEQ), which are DHA- and EPA-derived epoxy metabolites, were quantified in plasma. All results are shown as the mean ± SEM. Differences between the groups are significant as indicated (* *p* < 0.05; ** *p* < 0.01).

Epoxyeicosatrienoic acids (EETs), AA-derived epoxy metabolites synthesized by CYP450, were not affected in the different *n*-3 PUFA treatment groups (Figure 4A). Of the five CYP-dependent regioisomeric epoxyeicosatetraenoic acids (EEQ), we only measured 14,15-EEQ in plasma. *n*-3 PUFAs in the higher dose led to a significant increase in this metabolite (Figure 4C).

Epoxydocosapentaenoic acids (EDPs) are DHA-derived epoxy metabolites formed by cytochrome P450 monooxygenases [26]. EDPs are more potent than epoxyeicosatrienoic acids (EETs) regarding vasodilation [10] and anti-inflammation. In this study, supplementation with EPA and DHA in the higher dose elevated the levels of all examined EDP regioisomers (10,11-, 16,17-, and 19,20-EDP) (Figure 4B).

**Figure 5:**
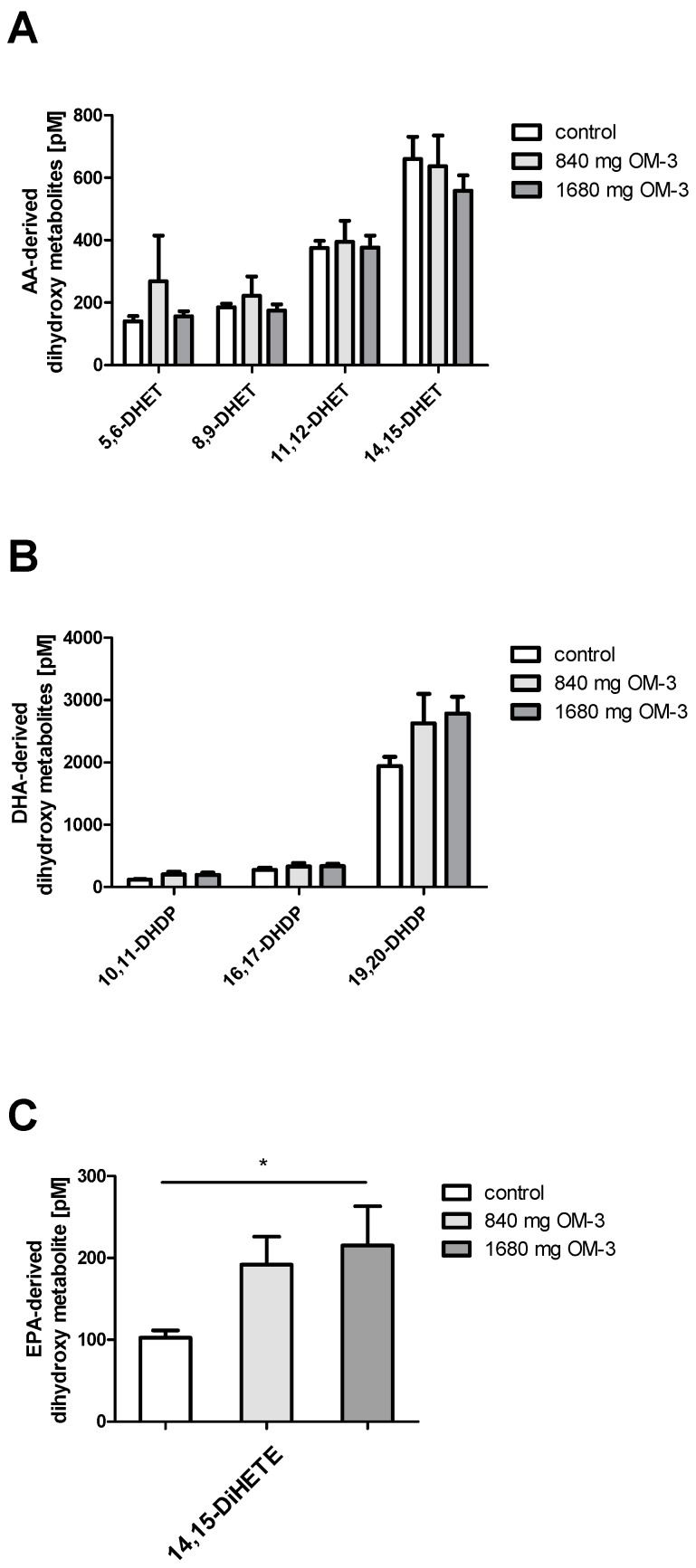
Profile of AA-, DHA- and EPA-derived dihydroxy metabolites. The dihydroxy metabolites are generated by the action of soluble epoxide hydrolase (sEH) from their parent epoxide metabolites. (**A**) Mean plasma levels of dihydroxyeicosatrienoic acids (DHETs), AA-derived dihydroxy metabolites are shown; (**B**) Mean plasma levels of dihydroxydocosapentaenoic acids (DHDPs), DHA-derived dihydroxy metabolites, are illustrated; (**C**) Mean plasma level of 14,15-DiHETE, an EPA-derived hydroxy metabolite, in correlation with different doses of *n*-3 PUFA is shown. All results are shown as the mean ± SEM. Differences between the groups are significant as indicated (* *p* < 0.05).

The action of, for example, the soluble epoxide hydrolase (sEH) converts the epoxy metabolites to the corresponding dihydroxy acids. Supplementation with EPA and DHA in subjects in this study did not affect the plasma levels of any of the dihydroxyeicosatrienoic acid (DHET) regioisomers assayed such as 5,6-, 8,9-, 11,12-, and 14,15-DHET (Figure 5A). As opposed to their parent epoxides, no effect of EPA and DHA supplementation on DHA-derived dihydroxydocosapentaenoic acids (10,11-, 16,17- and 19,20-DHDP) could be observed (Figure 5B). 14,15-Dihydroxyeicosatetraenoic acid (14,15-DiHETE), which is the corresponding vicinal diol of 14,15-EEQ, increased significantly with 1680 mg/day *n*-3 PUFA supplementation, similarly to its precursor epoxide (Figure 5C).

## 3. Discussion

To our knowledge, this is the first study to investigate the impact of a routine medication with *n*-3 PUFA in two different doses on AA-, DHA- and EPA-derived lipid metabolites in severely hyperlipidemic patients with atherosclerotic disease and the full spectrum of concomitant medications most notably aspirin and statins.

In hyperlipidemic patients without *n*-3 PUFA supplementation, the percentage of EPA and DHA of total fatty acids in blood cells was 3.6%, which is less than the value of 4.3% reported for healthy individuals [27]. Supplementation with EPA and DHA resulted in a substantial enrichment of these fatty acids, leading to an increase of the percentage to 6% in hyperlipidemic patients with a daily intake of 1680 mg *n*-3 PUFA. In comparison to the study conducted by Cao et al. [27], which showed an elevation from 4.3% to 7.8% in nine healthy subjects who ingested 1296 mg EPA and 864 mg DHA per day over 8 weeks, our hyperlipidemic patients were not able to reach the same amount as seen in these healthy subjects. This indicates that hyperlipidemic patients might need higher doses of EPA and DHA to reach the 8% recommended by Harris and von Schacky as cardioprotective [28].

Several mechanisms of the beneficial effects of *n*-3 PUFAs have been discussed: involving modulation of gene expression, interaction with membrane structures and ion channels, and alterations in eicosanoid biosynthesis. Omega-3 (*n*-3) PUFAs compete with AA at the *sn*-2 position of membrane phospholipids, thus interfering with AA metabolism [29] by providing alternative substrates for the enzymes of the LOX and COX and CYP450 families.

To evaluate the usage of these fatty acids for oxylipin formation, we measured various AA-, EPA-, and DHA-derived lipid mediators in blood plasma. We observed a 2.2-fold increase of 18-HEPE upon supplementation with 840 mg *n*-3 PUFA. Similarly, we found a marked increase in CYP-dependent epoxy metabolites of EPA (EEQ) and DHA (EDP) due to treatment with 1680 mg *n*-3 PUFA, while the corresponding AA-derived epoxy metabolites (EET) remained unaffected. The plasma level of 19,20-EDP, which has anti-thrombotic [30] and potent vasodilatory activity [10], increased 2.7 fold. EPA- and DHA-derived epoxy metabolites increased in a dose-dependent manner. While we observed a striking susceptibility of epoxy metabolites to *n*-3 PUFA treatment in hyperlipidemic patients, their corresponding oxidation products, the vicinal diols, remained unaffected.

In contrast to cytochrome P450-derived metabolites, the effects of EPA and DHA supplementation on 5-, 12-, and 15-LOX-dependent metabolites were almost not detectable with the exception of 5-HEPE, a 5-LOX-dependent EPA-derived metabolite. 17-HDHA (the precursor of the D-resolvin family) and 14-HDHA (the pathway indicator of anti-inflammatory maresins) were not significantly higher in patients treated with *n*-3 PUFAs, diverging from the results obtained in healthy individuals [11,12,13,14,15,16,31]. Other factors such as saturation or inhibition of the LOX and COX enzymes at higher *n*-3 PUFA doses might limit oxylipin formation in these pathways [32]. Alternatively, *n*-3 PUFA medication itself might lead to a downregulation of COX and LOX enzymes or, due to an anti-inflammatory effect [7], to lower levels of COX and LOX enzyme-expressing immune cells, thereby leading to reduced lipid mediator formation.

Concomitant treatment with statins in these hyperlipidemic patients might also limit the formation of particularly COX metabolites due to statin-triggered S-nitrosylation and acetylation of COX-2 and subsequent inactivation of the enzyme [33]. Statins could also blunt the production of *n*-3 PUFA-derived metabolites in hyperlipidemic patients by inhibiting 15-LOX, as shown in human endothelial cells [34].

Taken together, our data indicate that DHA and EPA can serve as alternative substrates for the LOX and CYP450 enzyme systems in hyperlipidemic patients, generating more anti-inflammatory, anti-thrombotic, and anti-oxidative metabolites while pro-inflammatory and oxidative stress-inducing mediators such as 12-LOX-derived metabolites [35] remain largely unchanged by the treatment.

However, a limitation of our study in this context is the lack of data regarding inflammation and liver function parameters in our assessment of the patients. This–combined with more comprehensive fatty acid and oxylipin analyses in the context of different standard medications (with and without aspirin and/or statin treatment)–will be part of larger future studies.

Further limitations of this study are due to its small nature and the assessment of *n*-3 PUFA supplementation that was merely based on a routine clinical treatment setting in a very selected patient population. On the other hand, a particularly interesting aspect of our study is that the assessment of *n*-3 PUFAs and their oxylipin lipidome was performed in the context of other cardiovascular co-medications, most notably aspirin and statins. Both have been implicated in the effects leading to the modification of lipid mediator formation. Future studies are required to help determine the adequate dose and duration of *n*-3 PUFA supplementation, taking into account that the activation of anti-inflammatory responses might be dependent not only on the dose of supplemented DHA and EPA but also on concomitant medication with drugs that interfere with (enhance/modulate/suppress) the spectrum of lipid mediators formed.

## 4. Materials and Methods

### 4.1. Patients and Blood Sampling

Patients of this cross-sectional study were recruited in the lipid outpatient department, Charité University Medicine Berlin, Campus Virchow. Thirty-five patients with hyperlipidemia undergoing long-term weekly extracorporeal lipoprotein apheresis to reduce levels of LDL-C and Lp(a) were included in this study. Please refer to specific papers for more information regarding lipid apheresis (e.g., [36]). Eleven patients received steady-state treatment with one capsule OMACOR^®^ (Pronova BioPharma NorgeAS, Sandefjord, Norway) per day (840 mg *n*-3 PUFA containing 460 mg EPA and 380 mg DHA as ethyl esters) and eight patients received treatment with two capsules OMACOR^®^ (1680 mg *n*-3 PUFA) per day, as part of their standard medical treatment according to the treating physician’s judgment. All patients received at least three months of prescription-grade omega-3 supplement, which can be considered to be sufficient to reach steady state values [11]. Sixteen apheresis patients without prescription-grade omega-3 supplementation served as controls. Generally, all subjects were advised to consume a diet low in both saturated fats and refined carbohydrates. The study was performed in accordance with the Declaration of Helsinki, current institutional guidelines, and good clinical practice. Our Institutional Review Board approved the study (approval code: EA2/061/12; approval date: 27 September 2012). After written informed consent, blood was drawn directly before an individual’s weekly lipid apheresis treatment. Plasma levels of total cholesterol, LDL-cholesterol, HDL-cholesterol, and triglycerides were determined with a photometrical method using a Roche fully-automated analyzer at the central routine laboratory facility of the Charité University Medicine in Berlin. For the analysis of PUFA and oxylipins, EDTA-blood was sampled directly before an individual’s lipid apheresis session. Plasma was obtained by centrifugation for 10 min at 4 °C and 1000× *g*. The lower (cellular) phase after centrifugation (predominantly red blood cells) was collected for the analysis of fatty acid composition by means of GC-FID. Supernatants were aliquoted and kept at −80 °C until analysis of lipid metabolites by LC-MS/MS.

### 4.2. Determination of Fatty Acid Pattern

From each sample, 50 µL of the cellular fraction was extracted with methyl-tert-butyl ether/methanol (MTBE/MeOH) and transesterified using methanolic hydrogen chloride. Fatty acid methyl esters (FAME) were quantified by gas chromatography-flame ionization detection (GC-FID) [37]. Samples for fatty acid analysis were analyzed within 7 days. In QC plasma samples, the major fatty acids (>0.25% of total FA) were quantified with an intraday and interday variance of ≤11%.

### 4.3. Sample Preparation and LC/ESI-MS/MS

Extraction of oxylipins from 500 µL human plasma was carried out as described [38]. Briefly, after addition of internal standard and antioxidant solution, samples were diluted with the same volume of methanol/water (5/95, *v*/*v*) acidified with 0.1% acetic acid, and extracted on Oasis HLB-SPE-cartridges (3 mL, 60 mg, 30 µm particles; Waters, Eschborn, Germany) using 0.5 mL methanol followed by 1.5 mL ethyl acetate for elution. LC-MS analysis of oxylipins was carried out as described [38]. Lipid mediators and deuterated standards used in this study were purchased from Biomol, Germany. Samples for oxylipin analysis were prepared within 1 day and analyzed within 3 days. In QC plasma samples, 74 analytes exceeded LLOQ and the intraday variance of most analytes (84% of quantified analytes) was <15%. Intraday variance for all 74 oxylipins was ≤30%. Concentrations of oxylipins are shown as pmol/L in plasma.

### 4.4. Statistical Analysis

Statistical analysis was performed using GraphPad Prism Software (La Jolla, CA, USA). Comparisons were carried out as one-way analysis of variance (ANOVA) with acceptance of statistical significance at *p* < 0.05. The data are represented as the mean ± standard error of the mean (SEM).

## Figures and Tables

**Table 1:** Characteristics of participants.

Parameter	Control (*n* = 16)	840 mg OM-3 (*n* = 11)	1680 OM-3 (*n* = 8)
Age	57 (42–71)	56 (43–73)	62 (52–72)
Body weight (kg)	89 (72.1–113.6)	91.1 (76.5–110.5)	80 (64–108)
BMI (kg/m^2^)	28.9 (24.1–35.5)	30.4 (25.4–39.2)	27.8 (22.3–30.3)
Lipid Parameters			
Total cholesterol (mg/dL)	180.8 (104–243)	178.4 (86–268)	226.4 (108–461)
LDL (mg/dL)	111.8 (52–281)	107.6 (41–146)	147.1 (52–332)
HDL (mg/dL)	48.9 (26–86)	53.4 (36–108)	45 (33–71)
LDL/HDL quotient	2.3	2.0	3.3
TG (mg/dL)	143.4 (57–281)	145.6 (84–247)	261.6 (123–490)
Medication			
Aspirin	16	11	8
Clopidogrel	6	3	2
Prasugrel	0	1	1
β-blockers	9	9	8
Statins	15	9	5
Simvastatin	9	5	4
Atorvastatin	4	3	0
Ezetimibe	11	6	3
Cardiovascular disease			
History of CAD	16	11	8
Arterial hypertension	10	11	6

Data are expressed as mean and range or number; OM-3, EPA/DHA-supplement as described in Materials and Methods; BMI, body mass index; CAD, coronary artery disease necessitating percutaneous transluminal coronary angioplasty (with stenting) or coronary artery bypass grafting; LDL, low-density lipoprotein cholesterol; HDL, high-density lipoprotein cholesterol; TG, triglycerides.

## Supplementary Materials

The following are available online at www.mdpi.com/1422-0067/19/1/180/s1.

## Abbreviations

LA	linoleic acid
HODE	hydroxyoctadecadienoic acid
EpOME	epoxyoctadecenoic acid
DiHOME	dihydroxyoctadecenoic acid
TriHOME	trihydroxyoctadecenoic acid
HETE	hydroxyeicosatetraenoic acid
EET	epoxyeicosatrienoic acid
DHET	dihydroxyeicosatrienoic acid
HOTrE	hydroxyoctadecatrienoic acid
EpODE	epoxyoctadecadienoic acid
DiHODE	dihydroxyoctadecadienoic acid
HEPE	hydroxyeicosapentaenoic acid
EEQ	epoxyeicosatetraenoic acid
DiHETE	dihydroxyeicosatetraenoic acid
HDHA	hydroxydocosahexaenoic acid
EDP	epoxydocosapentaenoic acid
DHDP	dihydroxydocosapentaenoic acid
