# Supplementary material for: Effect of Omega-3 Fatty Acid Supplementation on Oxylipins in a Routine Clinical Setting
Source: Int J Mol Sci. 2018 Jan 8;19(1):180. doi: 10.3390/ijms19010180 (PMC5796129; doi:10.3390/ijms19010180)
Supplement: Supplementary file 1 [file ijms-19-00180-s001.pdf]

Supplementary

Table S1. Fatty acid composition in the blood cell fraction of hyperlipidemic patients.

	Control group [µmol/L ± SEM]	840 mg OM-3 [µmol/L ± SEM]	1680 mg OM-3 [µmol/L ± SEM]
**PA (16:0)**	1582.42 ± 54.42	1633.69 ± 46.99	1725.11 ± 81.38
**SA (18:0)**	833.79 ± 30.88	867.63 ± 36.83	907.68 ± 49.26
**OA (18:1 n-9)**	915.20 ± 53.13	975.53 ± 48.75	980.77 ± 64.13
**LA (18:2 n-6)**	563.78 ± 26.58	544.44 ± 44.09	640.76 ± 51.46
**AA (20:4 n-6)**	587.13 ± 19.76	563.04 ± 31.56	540.55 ± 28.22
**EPA (20:5 n-3)**	28.87 ± 6.96	59.38 ± 5.74	101.72 ± 18.77
**DPA (22:5 n-3)**	35.36 ± 4.2	54.46 ± 7.72	58.49 ± 5.26
**DHA (22:6 n-3)**	115.35 ± 9.94	127.56 ± 10.82	166.74 ± 13.17

PA, palmitic acid; SA, stearic acid; OA, oleic acid; LA, linoleic acid; AA, arachidonic acid; EPA, eicosapentaenoic acid; DPA, docosapentaenoic acid; DHA, docosahexaenoic acid. OM-3, EPA/DHA-supplement as described in Materials and Methods.

Table S2. Concentration of oxylipins in plasma of hyperlipidemic patients.

	Control group [pmol/L ± SEM]	840 mg OM-3 [pmol/L ± SEM]	1680 mg OM-3 [pmol/L ± SEM]
	**LA-derived oxylipins**		
9-HODE	59052.1 ± 19795.1	76667.2 ± 37393.1	38643.1 ± 15215.1
13-HODE	53497.3 ± 20321	62318.1 ± 35122	31900.1 ± 15694.6
9,10-EpOME	2529.4 ± 839.6	7069.7 ± 5439.8	2532.8 ± 868.1
12,13-EpOME	7534.4 ± 2832.7	6563.8 ± 2602.8	6486.4 ± 2207.6
9,10-DiHOME	11162.5 ± 3678.6	24499.2 ± 18027.9	5608.9 ± 2022.1
12,13-DiHOME	21682.1 ± 5360.8	16077.6 ± 4247.6	10669.8 ± 2739
9,12,13-TriHOME	16054.4 ± 3812.6	16529.2 ± 3890.5	11447.1 ± 2697.1
9,10,13-TriHOME	967.3 ± 207.5	931.4 ± 150.6	893.6 ± 192.3
	**AA-derived oxylipins**		
5-HETE	552.2 ± 46.8	744.4 ± 171.6	517 ± 63.1
11-HETE	190.1 ± 16.9	201.9 ± 33.4	157.9 ± 12.3
12-HETE	542.5 ± 55.8	930.4 ± 425.3	557 ± 132.3
15-HETE	611 ± 38.7	713.9 ± 99.5	562.7 ± 59.9
20-HETE	449.5 ± 46.3	488.3 ± 64	477.6 ± 53.8
5,6-EET	446.9 ± 39.9	543.5 ± 100.6	734.1 ± 208.8
8.9-EET	59.3 ± 6.5	82.4 ± 21.2	81.4 ± 18.5
11,12-EET	112.3 ± 9.4	117 ± 17	151.2 ± 32.1
14,15-EET	114.2 ± 8.3	116.7 ± 16.9	142.6 ± 24.8
5,6-DHET	140.5 ± 17.4	269.1 ± 145.9	156.2 ± 16.8
8,9-DHET	184.8 ± 12.4	221.9 ± 62.1	174.9 ± 20.1
11,12-DHET	375 ± 23.4	395.2 ± 67.5	376.7 ± 38.1
14,15-DHET	660.3 ± 71.5	637 ± 98.8	559.2 ± 49.1
	**ALA-derived oxylipins**		
9-HOTrE	6621.5 ± 1537.5	5713.1 ± 1915.9	4093.1 ± 1796.1
13-HOTrE	12410.1 ± 2626.4	8631.4 ± 2561.1	6089.6 ± 2718.6
9,10-EpODE	120.8 ± 32.2	112.1 ± 24.6	199.9 ± 93.9
12,13-EpODE	449.3 ± 85.7	420.3 ± 110.1	354.9 ± 88.6
9,10-DiHODE	633.6 ± 176.1	415.2 ± 95.7	363.7 ± 124.6
12,13-DiHODE	659.3 ± 91.7	589.4 ± 121.8	433.9 ± 66.4
15,16-DiHODE	30269.8 ± 4935.5	27965.2 ± 6467.6	35129.9 ± 16536.8

**EPA-derived oxylipins**			
5-HEPE	197.2 ± 28.7	374.8 ± 76.6	340.1 ± 65.2
8-HEPE	97.9 ± 7.5	140.7 ± 27.4	154.5 ± 28.8
12-HEPE	186 ± 42.9	452.5 ± 249.3	307.2 ± 82.6
15-HEPE	182.4 ± 33.7	221.9 ± 29.8	223.7 ± 46.1
18-HEPE	869.3 ± 101.5	1924.41 ± 376.6	1482.43 ± 399.8
14,15-EEQ	36.3 ± 3.6	53.7 ± 7.4	93.5 ±32
8,9-DiHETE	81.5 ± 9.6	201.3 ± 57.1	180.6 ± 41.1
11,12-DiHETE	53.2 ± 6	134.6 ± 35.2	140.4 ± 33.9
14,15-DiHETE	102.7 ± 9	191.6 ± 34.6	215.2 ± 47.8
17,18-DiHETE	541.7 ± 44.3	1064.8 ± 220.2	1157.8 ± 198
**DHA-derived oxylipins**			
4-HDHA	294.6 ± 41.1	462 ± 74.8	477.2 ± 125.5
7-HDHA	51.4 ± 6.6	90 ± 17.2	84.4 ± 19.4
8-HDHA	353.9 ± 50.1	656.6 ± 109.9	598.3 ± 133.1
10-HDHA	90.8 ± 11.4	166.7 ± 24.1	156.6 ± 36.3
11-HDHA	115.7 ± 20.9	315.1 ± 131	214.4 ± 60.9
13-HDHA	74 ± 9.3	125.9 ± 21.4	129.9 ± 33.8
14-HDHA	428.8 ±101.2	1165.6 ± 704.5	664.1 ± 169.7
16-HDHA	112.8 ±10.7	178.9 ± 30	181.2 ± 39
17-HDHA	507.3 ± 61.5	810.03 ± 138.2	764.86 ± 138.5
20-HDHA	262.8 ± 29	426.9 ± 69.1	404.4 ± 82.8
10,11-EDP	101.4 ± 14.8	183.3 ± 42.9	300.8 ± 41.6
16,17-EDP	47.6 ± 4.9	80.1 ± 11.1	136.4 ± 51.5
19,20,-EDP	114.4 ± 16.4	215.1 ± 41.7	303.9 ± 93.6
4,5-DHDP	537.4 ± 49.6	648.8 ± 138.8	924.1 ± 177.6
10,11-DHDP	118.9 ± 10.5	205.1 ± 40.5	195.2 ± 39
13,14-DHDP	152.5 ± 11.1	220.9 ± 31.9	246.2 ± 35.1
16,17-DHDP	273.9 ± 35.4	331.6 ± 57.5	334.1 ± 40.7
19,20-DHDP	1904.3 ± 150.4	2628.2 ± 469.6	2784 ± 269

LA, linoleic acid; HODE, hydroxyoctadecadienoic acid; EpOME, epoxyoctadecenoic acid; DiHOME, dihydroxyoctadecenoic acid; TriHOME, trihydroxyoctadecenoic acid; AA, arachidonic acid; HETE, hydroxyeicosatetraenoic acid; EET, epoxyeicosatrienoic acid; DHET, dihydroxyeicosatrienoic acid; ALA, alpha-linolenic acid; HOTrE, hydroxyoctadecatrienoic acid; EpODE, epoxyoctadecadienoic acid; DiHODE, dihydroxyoctadecadienoic acid; EPA, eicosapentaenoic acid; HEPE, hydroxyeicosapentaenoic acid; EEQ, epoxyeicosatetraenoic acid; DiHETE, dihydroxyeicosatetraenoic acid; DHA, docosahexaenoic acid. HDHA, hydroxydocosahexaenoic acid; EDP, epoxydocosapentaenoic acid; DHDP, dihydroxydocosapentaenoic acid.
